# Destitute Workmen's Aid Society

**Published:** 1890-01-18

**Authors:** 


### Destitute Workmen's Aid Society.

The Metropolitan Destitute Workmen's Aid Society has been in existence only for some three years, and has already greatly improved the plan of helping the poor with which it started. This may be regarded as a fair indication that future amendments of its scheme will be adopted as experience shows the best lines upon which to advance. The society professes to be established to provide temporarily the deserving poor (not worthless vagrants), during sickness or slackness of employment, with free meals and lodgings by a system of tickets, honoured all over the metropolis, which are distributed only through recognised charitable workers and friends of the poor. Whatever we may think of this project as it stands, it is at least miles in advance of the original design, which was something like this, to enable everyone to be his own almoner at the smallest possible expenditure of money, and without any expenditure at all of thought, discrimination, or real care for the poor. Under the old plan a charitably-disposed person could write for a guinea's worth of very cheap tickets, and then silence every importunity in the streets with one or more of these tickets. In this way a sovereign was made to cover three or four times as many applications as could be disposed of for a like sum through gifts in cash. We all know that many are willing to give something to the poor, and that only comparatively few are willing to take trouble to see that the right people are helped in the best way. Without at all intending it, the first efforts of this society must have assisted in perpetuating very mischievous methods of poor relief. Time-honoured abuses die hard. Reform has cost the society some loss of income. The committee, however, has taken a firm stand in the matter, and will remember, we hope, that it would be better to dissolve the society than to return to the old system.

If the plan of relief by ticket is adopted at all, it is a good principle to give the tickets for distribution only to known workers amongst the poor, whose judgment and discrimination have been fully ascertained. This is the plan now followed, and the results, we have been assured, are satisfactory. Progressive philanthropists rather despise tickets as a medium of relief. Tickets have often been misused, sometimes sold for a copper or two, sometimes carefully deposited in the donor's letter-box. And so it has come to be felt that if a man is worthy of relief at all, he must be a person who can be safely entrusted with money. To this it is added that it is almost "impertinent relief" to tell a poor man the things he must get in a cookshop, instead of leaving him to decide the matter for himself. These opinions are not without weight, but are still only one side of the question. It may be replied, for instance, that a person who would use money well would use a ticket well. It also happens that relief must be given in many cases to persons who have to be saved from themselves, persons who would go to the public-house with money, but to the butcher or the baker with a ticket. This society has two additional safeguards: the money value of the ticket is small enough to make it unsaleable, and the people who distribute the tickets can be relied upon to give them only to those who will make a good use of them. Anyhow, there is a great array of testimony from competent workers that the society's tickets are a real help and benefit to the poor, and it is to the credit of the committee that they have "refused money" rather than depart from the principle of giving relief only through workers whose relations with the poor they have fully ascertained.

Lockhart's cocoa-rooms, and more than 200 coffee-houses, refreshment-rooms, etc., honour the tickets of the society, are said to be in sympathy with its work, and deal very liberally with those who bring tickets. The business-like action of the secretary goes far towards securing a welcome reception and generous treatment for these poor people. It is known that when the tickets are sent to headquarters, payment will be received by return of post. This contrasts most favourably with customs we have known to prevail—customs of unnecessarily long delay, sometimes extending over twelve months, in meeting the cost of tickets which shopkeepers have instantly honoured on the strength of the signature of the local clergyman, or someone else acting for the poor. The secretary regulates the bounty of the society by the amount of money in bank, a prudence for which the treasurer is, no doubt, grateful to him.

The sisters in hospitals receive some of these tickets, and are asked to use their discrimination in giving them to discharged patients. This is, perhaps, one of the best methods of relief adopted by the society. A patient is sometimes ordered, when leaving the hospital, not to resume work for a fortnight or a month, and any help towards his maintainence during that time is a real assistance in restoring him to complete health. We were glad to find the sisters in cordial co-operation with the society, and the society, on its part, fully appreciating the good use which they would make of its benefactions.

In the matter of loans, this society has also advanced a step. Formerly, loans were given on recommendation; now they are given on personal responsibility undertaken in writing. The society avoids loss by the present method, and also brings into its work people who do really interest themselves in the poor, and have learned enough about their clients to judge of the wisdom of being responsible for the loans they receive. Dishonest applications are thus reduced, or made impossible, and honest poor men are discouraged from undertaking obligations which they never could fulfil.

To say anything about the lodging-houses to which the London poor resort would introduce a large and complicated subject. People will hear more by-and-by of a large scheme in which the Bishop of Marlborough has specially interested himself, and which will seek to purify the lodging-house system without increasing the charges to the poor. The Destitute Workmen's Aid Society has selected the most respectable lodging-houses it could find in different parts of London, and arranged with those who have charge of them to lodge persons holding the society's tickets. Only one lodging-house for women appears in the society's list, and of this there is a twofold explanation: it is hard to find thoroughly suitable houses of this class, and the various refuges which are to be found are our best apology for this difficulty.

We were not able to secure successive annual reports, and to estimate, from such official sources, the progress of this society. On its old footing, there seems to have been an income of something over £600 a year. We cannot say how much of this has been withdrawn by lovers of antiquated abuses in the matter of charitable relief, or what new subscriptions may have come from those desiring reform and advance.

We do not pretend to be sanguine even as to the future of this organisation, and have freely declared our mind as to its past efforts. Whilst admiring the good and benevolent intentions of those who have founded and carried it on, we cannot help regarding the whole plan as reactionary both in design and execution. Certainly it is not an institution born of the spirit of our own times, but seems rather to have sprung from opposition to principles which now more and more prevail. It offers temporary relief where the need must often be a permanent one, it is the old idea of small doles (very small) in full force once more, and it is relief in food, and by ticket, instead of in cash or by providing work for those desiring employment. It seeks, we fear, to perform a task which is all but impossible in a quite impossible way. If the society's income could be counted by thousands instead of hundreds of pounds, would it even then be possible to feed the half-starving thousands in London by occasional and small meals at entirely uncertain intervals? If we must have one more organisation, with the increased possibilities it brings of overlapping the work which others are doing, it would be better, we think, if a less ambitious field of operation were selected. In a prescribed area, with full knowledge of the poor, and of the work performed by other agencies therein, £600 might be annually expended in a really effective way, in a manner to secure permanent benefit to those assisted. But this, or, indeed, any sum, devoted to supplying mouthfuls of food now and again to starving people all over London, cannot materially assist what we must all wish to see accomplished—some certain provision in each locality for the particular needs of the poor living and struggling there.

